# Urinary heavy metal burden and overactive bladder risk: a cross-sectional study based on NHANES 2005–2018

**DOI:** 10.1097/JS9.0000000000003214

**Published:** 2025-08-19

**Authors:** Jinbao Wang, Qiyou Wu, Bo Chen, Jinze Li, Jinjiang Jiang, Xinyang Liao, Qiang Wei

**Affiliations:** a Department of Urology, West China Hospital, Sichuan University, Chengdu, Sichuan Province, China; b West China School of Medicine, Sichuan University, Chengdu, Sichuan Province, China

**Keywords:** heavy metal, NHANES, OAB, relationship, WQS

## Abstract

**Objective:**

We aimed to explore the relationship between urinary heavy metals and overactive bladder (OAB) among US adults.

**Method:**

Data were obtained from the National Health and Nutrition Examination Survey 2005–2018. Concentrations of heavy metals in urine were determined by laboratory tests and corrected for urinary creatinine using natural logarithm (ln) transformation. OAB was assessed using questionnaires. The association between each urinary heavy metal and OAB was first explored using multivariate logistic regression, followed by nonlinear correlation analyses and smoothed curve fitting, with stratified analyses and interaction tests for age and sex. In addition, weighted quantile sum (WQS) regression and quantile-based g-computation (qgcomp) analyses were implemented to explore the relationship between mixed heavy metal and OAB.

**Results:**

A total of 9086 participants were included in the final study. Multifactorial logistic regression analysis showed that cobalt (Co), lead (Pb), cadmium (Cd), and uranium (Ur) were positively associated with the risk of developing OAB, whereas barium (Ba) and thallium (Tl) were negatively associated with OAB. Nonlinear correlation analysis showed that the associations between the concentrations of Ba and Tl and OAB risk were nonlinear, and Pb, antimony (Sb), tungsten (Tu), Ur, and Cd showed significant positive correlations with OAB. WQS regression showed that OAB risk increased with increasing quartiles of the WQS index [odds ratio (OR) 1.202, 95% confidence interval (CI) 1.064–1.357], with Cd showing the strongest positive correlation with OAB. When the mixture effect was constrained to be negative, an increase in the WQS index quartile corresponded to a 14.3% reduction in OAB risk (OR 0.857, 95% CI 0.778–0.943), with Ba standing out as the most dominant. The results of qgcomp analysis showed that Cd had the largest positive weight of 0.5098, and Ba had the largest negative weight of 0.5976.

**Conclusion:**

Urinary heavy metals are significantly associated with OAB risk among US adults. The metal contributing most to the positive correlation with OAB risk is Cd, while the metal contributing most to the negative correlation with OAB risk is Ba.

## Introduction

Overactive bladder (OAB) is a syndrome of storage symptoms characterized by urgency, sometimes with urge urinary incontinence (UUI), along with urinary frequency and nocturia^[1]^. In a survey of five countries in Europe and the United States, the prevalence of OAB in the overall population was 11.8%, with insignificant differences between males and females, and increased with age^[2]^. OAB adversely affects quality of life and may increase risk of falls and fractures, sleep disorders, and depression in older adults^[3]^. Some studies have shown that the occurrence of OAB is associated with age, gender, obesity, and bowel symptoms^[4–6]^. In addition, it has been suggested that oxidative stress, metabolic syndrome (MetS), and alterations in sex hormones may also play roles in the pathogenesis of OAB^[7]^.

**HIGHLIGHTS**

- Used a variety of statistical methods to evaluate the effects of heavy metals.
- Urinary heavy metals are significantly associated with OAB risk.
- Cd shows the strongest positive association with OAB risk.
- Ba shows the strongest negative association with OAB risk.
- The relationship between some heavy metals and OAB risk is not nonlinear.

Heavy metals are typically defined as those with a density greater than 4.5 g/cm^3^. In the process of industrialization and urbanization, pollutants containing large quantities of heavy metals are produced^[8]^, and these metals enter the environment and can accumulate in the human body via the digestive tract, respiratory tract, and skin^[9]^. Overexposure to heavy metals leads to oxidative stress, causing effects such as DNA damage, lipid peroxidation, and protein modification^[10]^. This oxidative stress can induce a range of cellular responses associated with an increased risk of tumors, neurological disorders, and hormone-related diseases^[11]^. High levels of cadmium (Cd) and lead (Pb) (nephrotoxic metals) are associated with higher risk of urinary incontinence in women^[12]^. Previous studies have reported the association between heavy metal exposure and stress urinary incontinence^[13,14]^; however, they focused solely on stress urinary incontinence and did not specifically analyze UUI, a key component of OAB. To date, few studies have explored the relationship between multiple urinary heavy metal exposures and UUI, nocturia, or OAB.

Given that heavy metal exposure can induce responses such as oxidative stress and endocrine alterations, and considering the established links between OAB and sex hormones, as well as metabolism, it is biologically plausible that an association exists between OAB and heavy metal exposure. Therefore, the main objective of this study was to investigate the association between multiple urinary heavy metal exposures and OAB risk using diverse statistical methods and to identify the metals contributing most significantly to this risk. Through our study, we can provide a more comprehensive perspective on the influence of heavy metals on OAB, thereby contributing to the theoretical basis for environmental and health-related policy decisions.

## Methods

### Study cohort and population

The National Health and Nutrition Examination Survey (NHANES) is an ongoing program conducted by the US National Center for Health Statistics (NCHS) that covers a wide range of demographic, dietary, and medical issues. With a biennial cycle, these surveys focus on epidemiologic and health science research and are a critical resource for the assessment of the health and nutritional status of the US population.

In this study, we included data from seven survey cycles of NHANES 2005–2018 (2005–2006, 2007–2008, 2009–2010, 2011–2012, 2013–2014, 2015–2016, and 2017–2018). A total of 39 749 participants with data from the Kidney Conditions questionnaire were initially included. Subsequently, we excluded participants who (1) were younger than 20 years, (2) had missing information on urge incontinence or nocturia, and (3) had missing concentrations of urinary metals. A total of 9086 participants were included in the final analysis. NHANES data collection was approved by the Ethical Review Committee of the NCHS. All respondents signed informed consent.

### Measurements of metals

In NHANES, since most metal exposure concentrations were measured from urine samples, urinary metal concentrations in urine samples were chosen for our study as a way to comprehensively investigate the association between multiple metal exposures and OAB. Compared with blood metal measurement, urinary metal measurement is often preferred due to its noninvasive, rapid, and sensitive characteristics^[15]^. Urine sample analysis was performed at the Division of Laboratory Sciences, National Center for Environmental Health, Centers for Disease Control and Prevention, Atlanta, GA. Twelve urinary metallic elements were detected based on an analytical technique called inductively coupled plasma-mass spectrometry. Values below the limit of detection (LOD) were imputed as the LOD divided by √2. The experimental procedures and details of the analysis are available on the NHANES website (https://www.cdc.gov/nchs/nhanes/). Since over 80% of the measured values for beryllium and platinum were below the LOD^[16]^, we excluded them and included the following 10 metals in the study: barium (Ba), cobalt (Co), cesium (Cs), molybdenum (Mo), Pb, antimony (Sb), thallium (Tl), tungsten (Tu), uranium (Ur), and Cd. We corrected the concentrations of these 10 metals for urinary creatinine and expressed them with μg/g creatinine^[17]^. Due to the non-normal distribution of urinary heavy metal concentrations, natural logarithm (ln) transformation was applied for subsequent analysis to minimize distributional bias.

### Assessment of OAB

OAB was assessed using questionnaire items administered to participants that included both UUI and nocturia and was ultimately diagnosed based on the Overactive Bladder Symptom Score (OABSS)^[18]^. UUI was assessed with the question: “During the past 12 months, have you leaked or lost control of even a small amount of urine with an urge or pressure to urinate and you couldn’t get to the toilet fast enough?,” and when the respondent answered “Yes,” further follow-up questions were asked about the frequency. Responses of “Never,” “Less than once a month,” “A few times a month,” “A few times a week,” and “Every day and/or night” were assigned UUI scores of 0, 1, 1, 2, and 3, respectively. Nocturia was assessed by asking “During the past 30 days, how many times per night did you most typically get up to urinate, from the time you went to bed at night until the time you got up in the morning.” Participants answered “0,” “1,” “2,” “3,” “4,” and “5 or more,” and the resulting nocturia scores were 0, 1, 2, 3, 3, and 3, respectively. Subsequently, the two scores were summarized to obtain the OABSS, and those with a total score of ≥3 were classified as having OAB^[19–22]^.

### Covariates

In this study, we selected a number of covariates that may affect the results, including age, gender, ethnicity, and education level. All covariates were obtained through a questionnaire. The ratio of family income to poverty was used to reflect socioeconomic status and categorized as <1.3, 1.3–3.5, and >3.5. Education level included less than high school, high school or general educational development, and above high school. Marital status was categorized as married or living with partner and living alone. The comorbidity index, indicating comorbidity burden, was categorized as 0, 1, and ≥2 based on the total score. Alcohol intake was categorized as none, moderate, or heavy. Physical activity was categorized as less than moderate, moderate, or vigorous based on the intensity of activity. Additionally, some covariates had missing values (constituting less than 10% of the total data for each variable). We grouped the missing values as “other” for analysis, as shown in Table 1.

**Table 1:** Baseline characteristics of the participants

Exposure	Overall	Non-OAB	OAB	*P*value
Participants (*n*)	9086	7229 (79.56%)	1857 (20.44%)	
Age (year), mean ± SD	48.80 ± 17.61	46.37 ± 17.11	58.26 ± 16.26	<0.001
Gender, *n* (%)				<0.001
Male	4612 (50.76%)	3853 (53.30%)	759 (40.87%)	
Female	4474 (49.24%)	3376 (46.70%)	1098 (59.13%)	
Ethnicity, *n* (%)				<0.001
Mexican American	1333 (14.67%)	1065 (14.73%)	268 (14.43%)	
Other Hispanic	795 (8.75%)	639 (8.84%)	156 (8.40%)	
Non-Hispanic White	4225 (46.50%)	3452 (47.75%)	773 (41.63%)	
Non-Hispanic Black	1982 (21.81%)	1424 (19.70%)	558 (30.05%)	
Other Race	751 (8.27%)	649 (8.98%)	102 (5.49%)	
Ratio of family income to poverty, *n* (%)				<0.001
<1.3	2821 (31.05%)	2136 (29.55%)	685 (36.89%)	
1.3-3.5	3136 (34.51%)	2457 (33.99%)	679 (36.56%)	
>3.5	2484 (27.34%)	2142 (29.63%)	342 (18.42%)	
Other	645 (7.10%)	494 (6.83%)	151 (8.13%)	
Education level, *n* (%)				<0.001
Less than high school	2284 (25.14%)	1623 (22.45%)	661 (35.60%)	
High school or General educational development	2141 (23.56%)	1750 (24.21%)	391 (21.06%)	
Above high school	4419 (48.64%)	3656 (50.57%)	763 (41.09%)	
Other	242 (2.66%)	200 (2.77%)	42 (2.26%)	
Marital, *n* (%)				<0.001
Married or living with partner	5656 (62.25%)	4647 (64.28%)	1009 (54.33%)	
Living alone	3428 (37.73%)	2581 (35.70%)	847 (45.61%)	
Other	2 (0.02%)	1 (0.01%)	1 (0.05%)	
BMI, *n* (%)				<0.001
≤25	2765 (30.43%)	2340 (32.37%)	425 (22.89%)	
25–30	3024 (33.28%)	2490 (34.44%)	534 (28.76%)	
>30	3214 (35.37%)	2354 (32.56%)	860 (46.31%)	
Other	83 (0.91%)	45 (0.62%)	38 (2.05%)	
Comorbidity index, *n* (%)				<0.001
0	5176 (56.97%)	4529 (62.65%)	647 (34.84%)	
1	2792 (30.73%)	2016 (27.89%)	776 (41.79%)	
≥2	1032 (11.36%)	629 (8.70%)	403 (21.70%)	
Other	86 (0.95%)	55 (0.76%)	31 (1.67%)	
Smoking status, *n* (%)				<0.001
Never	4360 (47.99%)	3564 (49.30%)	796 (42.86%)	
Former	2314 (25.47%)	1766 (24.43%)	548 (29.51%)	
Current	2410 (26.52%)	1897 (26.24%)	513 (27.63%)	
Other	2 (0.02%)	2 (0.03%)	0 (0.00%)	
Alcohol intake, *n* (%)				<0.001
None	6475 (71.26%)	5031 (69.59%)	1444 (77.76%)	
Moderate	751 (8.27%)	630 (8.71%)	121 (6.52%)	
Heavy	1560 (17.17%)	1344 (18.59%)	216 (11.63%)	
Other	300 (3.30%)	224 (3.10%)	76 (4.09%)	
Physical activity, *n* (%)				<0.001
Less than moderate	4677 (51.47%)	3478 (48.11%)	1199 (64.57%)	
Moderate	1123 (12.36%)	924 (12.78%)	199 (10.72%)	
Vigorous	3225 (35.49%)	2792 (38.62%)	433 (23.32%)	
Other	61 (0.67%)	35 (0.48%)	26 (1.40%)	

BMI, body mass index; OAB, overactive bladder.

In addition, for females, two variables, parity and vaginal delivery history, were included in the sensitivity analyses. Parity was assessed by the question “How many deliveries live birth result?” whereas vaginal delivery was assessed by the question “How many vaginal deliveries?” For males, we included benign prostatic hyperplasia (BPH). Participants were asked, “Have you ever been told by a doctor or health professional that you had an enlarged prostate gland?” Those answering “Yes” were further asked if the enlargement was benign. Only those confirming benign enlargement were classified as having BPH.

We also obtained participants’ serum creatinine (mg/dL) and calculated their glomerular filtration rate (eGFR) using the following formula^[23,24]^: eGFR = 175 × Serum creatinine^−1.154^ × Age^−0.203^ × 0.742 (if female) × 1.212 (if Black). In this equation, eGFR is measured in mL/min/1.73 m^2^.

### Statistical analysis

Baseline characteristics of the study population are described in Table 1. Continuous variables are presented as the mean ± standard deviation, while categorical variables are shown as percentages. The Kruskal–Wallis rank sum test and chi-square test were used to calculate *P* values for continuous and categorical variables, respectively, and for counting variables, Fisher’s exact probability test was used to obtain *P* values if there was a theoretical number <10.

To explore the association between single urinary metals and OAB, the ln-transformed, creatinine-corrected urinary metal concentrations were categorized into quartiles based on their distribution to assess the effect of Q2, Q3, and Q4 on the prevalence of OAB compared with the Q1 group. The odds ratios (ORs) were the effect value of multivariate logistic regression. We constructed four models. Model 1 was not adjusted for any variables, and model 2 was adjusted for gender, age, ethnicity, ratio of family income to poverty, education level, and marriage. Model 3 was further adjusted for body mass index (BMI), comorbidity index, smoking status, alcohol intake, and physical activity, in addition to those adjusted for in model 2. We also assessed the role of urinary metal exposure as a continuous variable on outcomes. To exclude the effect of renal function on urinary heavy metal concentrations, given the formula for eGFR, we adjusted for all covariates in model 3 except age, gender, and ethnicity, with additional adjustment for eGFR.

Subsequently, we used generalized additive modeling and smooth curve fitting to test for nonlinear associations between each urinary metal concentration and OAB in the model adjusted for all covariates. We also carried out subgroup analyses and interaction tests by gender and age (dichotomized at 60 years) to examine potential effect modification by these factors in the association between urinary metals and OAB.

To evaluate the overall effect of mixed urinary metals on OAB and the importance of each metal, we performed weighted quantile sum (WQS) regression. This method estimates the combined effect of multiple exposures on an outcome by constructing a weighted index and detecting its association with the outcome^[25]^. Furthermore, by assigning weights to each exposure variable through the model, it assessed the significance of the impact of individual variables on the outcome^[25]^. A key feature of WQS regression is that it assumes all exposures in the mixture act in the same direction (either all positively or all negatively associated with the outcome). In our study, we performed a WQS regression based on the quantiled urinary metal concentrations (Q1–Q4) after adjusting for all covariates, with 40% of the data in the test set and 60% of the data in the validation set^[26–28]^. The model was set up with a total of 1000 bootstraps to estimate the weights. The correlation between urine metals and OAB was explored in two directions: positive and negative correlation.

In addition, we used quantile-based g-computation (qgcomp) to examine the relationship between OAB and mixed heavy metal exposures. qgcomp methods allow for the assessment of the overall effect of the exposure mixture on OAB by combining the quantiles^[29]^ exposures and do not necessitate the definition of directionality.

Sensitivity analyses were conducted to assess the potential influence of BPH in men, and parity and vaginal delivery history in women, on the observed associations. For both men and women, we constructed three separate models. None of the model 1 was adjusted in any way. For men, model 2 was adjusted for BPH, and for women, model 2 was adjusted for parity and vaginal delivery history. Model 3 was adjusted for age, ethnicity, ratio of family income to poverty, education level, marriage, BMI, comorbidity index, smoking status, alcohol intake, and physical activity based on model 2. Physical activity was adjusted. As in the primary analysis, ln-transformed urinary metal creatinine-corrected concentrations were analyzed both as continuous and categorized into quartiles.

Statistical analyses for our study were performed with the R software package (http://www.R-project.org, R Foundation) and EmpowerStats software (www.empowerstats.com). A two-sided *P* value <0.05 was considered statistically significant. Specifically, the WQS model was implemented using the gWQS package R. In addition, the work has been reported in line with the Revised Strengthening the reporting of cohort, cross-sectional and case-control studies in surgery (STROCSS) criteria^[30]^.

## Results

### Baseline characteristics

The baseline characteristics of the participants are presented in Table 1. A total of 9086 participants were finally included in our study, with a mean age of 48.80 ± 17.61 years and 50.76% males. A total of 1857 (20.44%) participants were classified as having OAB. In the overall population, non-Hispanic White, with education above high school, married or living with partner, never smoking and no alcohol intake were more prevalent. There were significant differences between the non-OAB and OAB populations in gender, age, ethnicity, ratio of family income to poverty, education level, marital status, BMI, comorbidity index, smoking status, alcohol intake, and physical activity. Compared to the non-OAB population, the OAB population had a higher proportion of females, a lower ratio of family income to poverty, lower educational attainment, higher BMI, more comorbidities, and lower levels of vigorous physical activity.

### Multivariate logistic regression

The results of the multivariate logistic regression are presented in Table 2. In model 1 without adjustment for covariates, creatinine-corrected and ln-transformed concentrations of Ba and Tl (as continuous variables) were negatively associated with OAB risk, while Co, Pb, Sb, Tu, Ur, and Cd were positively associated. After adjusting for covariates, the positive associations of Co, Pb, Sb, Ur, and Cd with OAB risk remained significant. In model 3 (fully adjusted), the risk of OAB increased by 29% [OR 1.29, 95% confidence interval (CI) 1.20–1.40], 18% (OR 1.18, 95% CI 1.09–1.27), and 15% (OR 1.15, 95% CI 1.08–1.22) for each 1-unit increase in the concentration of Cd, Pb, and Ur, respectively. When urinary metals were analyzed as quartiles, negative associations for Ba and Tl and positive associations for Co, Pb, Sb, Tu, Ur, and Cd with OAB risk were still observed. In model 1, participants in the highest quartile of Ba and Tl had a 22% (OR 0.78, 95% CI 0.68–0.90) and 14% (OR 0.86, 95% CI 0.75–0.99) lower risk of OAB compared to those in the lowest quartile, respectively. Notably, participants in the highest quartile of Cd, Pb, and Ur had 4.08 times (OR 4.08, 95% CI 3.45–4.82), 2.02 times (OR 2.02, 95% CI 1.74–2.35), and 1.72 times (OR 1.72, 95% CI 1.49–2.00) higher risk, respectively. In particular, for Cd, even in model 3 adjusted for all covariates, participants in the highest quartile still had a 2.09-fold higher risk of OAB than those in the lowest quartile (OR 2.09, 95% CI 1.72–2.55). In model 4, each unit increase in ln-transformed Pb and Cd concentrations still increased the risk of OAB by 31% (OR 1.31, 95% CI 1.20–1.42) and 51% (OR 1.51, 95% CI 1.40–1.64), respectively. These correlations remained significant after treating heavy metal concentrations as categorical variables.

**Table 2:** Multivariate regression models of the association between heavy metals and OAB risk

Metals	Continuous		Q2	Q3	Q4	*P* for trend
	OR (95% CI)	Q1	OR (95% CI)	OR (95% CI)	OR (95% CI)	
Ba						
Model 1	**0.88 (0.84, 0.93)**	1.00 (reference)	**0.78 (0.68, 0.90)**	**0.64 (0.55, 0.74)**	**0.78 (0.68, 0.90)**	**<0.0001**
Model 2	0.95 (0.90, 1.01)	1.00 (reference)	0.95 (0.81, 1.10)	**0.77 (0.66, 0.91)**	0.93 (0.80, 1.09)	0.1392
Model 3	0.99 (0.93, 1.05)	1.00 (reference)	1.00 (0.86, 1.17)	0.85 (0.73, 1.00)	1.03 (0.88, 1.21)	0.8673
Model 4	0.97 (0.92, 1.04)	1.00 (reference)	0.96 (0.82, 1.14)	**0.82 (0.69, 0.97)**	0.99 (0.84, 1.18)	0.5472
Co						
Model 1	**1.17 (1.09, 1.26)**	1.00 (reference)	1.05 (0.90, 1.22)	1.10 (0.95, 1.27)	**1.39 (1.21, 1.60)**	**<0.0001**
Model 2	**1.13 (1.04, 1.22)**	1.00 (reference)	1.07 (0.91, 1.26)	1.10 (0.93, 1.29)	**1.30 (1.10, 1.53)**	**0.0014**
Model 3	**1.12 (1.04, 1.22)**	1.00 (reference)	1.08 (0.92, 1.28)	1.08 (0.92, 1.28)	**1.30 (1.10, 1.54)**	**0.0018**
Model 4	**1.15 (1.05, 1.25)**	1.00 (reference)	1.02 (0.86, 1.22)	1.07 (0.90, 1.28)	**1.30 (1.09, 1.54)**	**0.0016**
Cs						
Model 1	1.06 (0.95, 1.17)	1.00 (reference)	0.92 (0.79, 1.06)	1.03 (0.89, 1.18)	1.09 (0.94, 1.26)	0.1193
Model 2	1.01 (0.89, 1.13)	1.00 (reference)	0.92 (0.79, 1.08)	1.01 (0.86, 1.19)	1.02 (0.86, 1.21)	0.5929
Model 3	1.07 (0.95, 1.20)	1.00 (reference)	0.95 (0.81, 1.11)	1.04 (0.88, 1.22)	1.11 (0.93, 1.31)	0.1536
Model 4	**1.15 (1.02, 1.30)**	1.00 (reference)	1.01 (0.85, 1.19)	1.14 (0.96, 1.35)	**1.24 (1.05, 1.48)**	**0.0060**
Mo						
Model 1	1.07 (1.00, 1.16)	1.00 (reference)	0.98 (0.85, 1.13)	1.01 (0.87, 1.17)	1.14 (0.99, 1.32)	0.0616
Model 2	1.03 (0.95, 1.12)	1.00 (reference)	0.98 (0.84, 1.14)	1.03 (0.88, 1.20)	1.05 (0.90, 1.23)	0.4533
Model 3	1.06 (0.97, 1.16)	1.00 (reference)	1.00 (0.86, 1.17)	1.04 (0.89, 1.22)	1.11 (0.94, 1.30)	0.1948
Model 4	1.07 (0.98, 1.18)	1.00 (reference)	1.04 (0.87, 1.23)	1.06 (0.89, 1.26)	1.15 (0.97, 1.36)	0.1170
Pb						
Model 1	**1.38 (1.29, 1.47)**	1.00 (reference)	**1.28 (1.09, 1.50)**	**1.70 (1.46, 1.98)**	**2.02 (1.74, 2.35)**	**<0.0001**
Model 2	**1.13 (1.04, 1.22)**	1.00 (reference)	1.06 (0.90, 1.25)	**1.24 (1.06, 1.47)**	**1.29 (1.09, 1.52)**	**0.0007**
Model 3	**1.18 (1.09, 1.27)**	1.00 (reference)	1.08 (0.91, 1.28)	**1.29 (1.09, 1.53)**	**1.39 (1.17, 1.65)**	**<0.0001**
Model 4	**1.31 (1.20, 1.42)**	1.00 (reference)	**1.32 (1.10, 1.59)**	**1.62 (1.35, 1.94)**	**1.79 (1.49, 2.15)**	**<0.0001**
Sb						
Model 1	**1.16 (1.08, 1.25)**	1.00 (reference)	1.09 (0.94, 1.27)	**1.24 (1.07, 1.43)**	**1.32 (1.14, 1.53)**	**<0.0001**
Model 2	**1.10 (1.01, 1.19)**	1.00 (reference)	1.03 (0.88, 1.21)	1.11 (0.95, 1.30)	**1.18 (1.01, 1.38)**	**0.0205**
Model 3	**1.13 (1.04, 1.23)**	1.00 (reference)	1.06 (0.90, 1.25)	1.14 (0.97, 1.34)	**1.23 (1.05, 1.45)**	**0.0068**
Model 4	**1.14 (1.05, 1.25)**	1.00 (reference)	1.05 (0.88, 1.25)	1.19 (1.00, 1.41)	**1.25 (1.05, 1.49)**	**0.0051**
Tl						
Model 1	**0.87 (0.79, 0.95)**	1.00 (reference)	**0.76 (0.66, 0.88)**	**0.73 (0.64, 0.85)**	**0.86 (0.75, 0.99)**	**0.0294**
Model 2	0.91 (0.82, 1.01)	1.00 (reference)	0.88 (0.75, 1.02)	**0.80 (0.69, 0.94)**	0.94 (0.80, 1.10)	0.2955
Model 3	0.97 (0.87, 1.07)	1.00 (reference)	0.91 (0.78, 1.06)	**0.83 (0.71, 0.97)**	1.03 (0.88, 1.21)	0.9265
Model 4	1.05 (0.94, 1.18)	1.00 (reference)	0.86 (0.72, 1.01)	0.86 (0.72, 1.02)	1.12 (0.95, 1.33)	0.2019
Tu						
Model 1	**1.07 (1.01, 1.14)**	1.00 (reference)	1.00 (0.86, 1.16)	1.00 (0.86, 1.16)	**1.20 (1.04, 1.38)**	**0.0134**
Model 2	1.06 (1.00, 1.13)	1.00 (reference)	0.96 (0.83, 1.13)	0.96 (0.82, 1.12)	**1.17 (1.00, 1.36)**	**0.0416**
Model 3	1.06 (1.00, 1.13)	1.00 (reference)	0.98 (0.83, 1.14)	0.96 (0.82, 1.13)	**1.17 (1.00, 1.37)**	**0.0450**
Model 4	1.06 (0.99, 1.13)	1.00 (reference)	0.95 (0.80, 1.13)	1.01 (0.85, 1.19)	1.16 (0.98, 1.38)	0.0543
Ur						
Model 1	**1.23 (1.17, 1.30)**	1.00 (reference)	**1.23 (1.06, 1.44)**	**1.38 (1.18, 1.60)**	**1.72 (1.49, 2.00)**	**<0.0001**
Model 2	**1.16 (1.09, 1.23)**	1.00 (reference)	**1.18 (1.00, 1.38)**	**1.23 (1.05, 1.45)**	**1.51 (1.29, 1.77)**	**<0.0001**
Model 3	**1.15 (1.08, 1.22)**	1.00 (reference)	**1.19 (1.01, 1.41)**	**1.23 (1.04, 1.45)**	**1.50 (1.27, 1.77)**	**<0.0001**
Model 4	**1.16 (1.09, 1.24)**	1.00 (reference)	**1.22 (1.02, 1.46)**	**1.25 (1.05, 1.49)**	**1.50 (1.26, 1.79)**	**<0.0001**
Cd						
Model 1	**1.71 (1.61, 1.81)**	1.00 (reference)	**2.00 (1.68, 2.39)**	**3.10 (2.62, 3.67)**	**4.08 (3.45, 4.82)**	**<0.0001**
Model 2	**1.33 (1.24, 1.43)**	1.00 (reference)	**1.63 (1.36, 1.96)**	**2.05 (1.71, 2.45)**	**2.30 (1.92, 2.76)**	**<0.0001**
Model 3	**1.29 (1.20, 1.40)**	1.00 (reference)	**1.51 (1.25, 1.83)**	**1.86 (1.54, 2.25)**	**2.09 (1.72, 2.55)**	**<0.0001**
Model 4	**1.51 (1.40, 1.64)**	1.00 (reference)	**1.69 (1.38, 2.07)**	**2.45 (2.01, 2.99)**	**2.97 (2.42, 3.65)**	**<0.0001**

Ba, barium; BMI, body mass index; Cd, cadmium; CI, confidence interval; Co, cobalt; Cs, cesium; eGFR, glomerular filtration rate; Mo, molybdenum; OAB, overactive bladder; OR, odds ratio; Pb, lead; Sb, antimony; Tl, thallium; Tu, tungsten; Ur, uranium.

OR (95% CI) for *P* values <0.05 are in bold.

Model 1 adjusted for none.

Model 2 adjusted for gender, age, ethnicity, ratio of family income to poverty, education level, and marital.

Model 3 adjusted for gender, age, ethnicity, ratio of family income to poverty, education level, marital, BMI, comorbidity index, smoking status, alcohol intake, and physical activity.

Model 4 adjusted for eGFR, ratio of family income to poverty, education level, marital, BMI, comorbidity index, smoking status, alcohol intake, and physical activity.

### Nonlinear correlation analysis

**Figure 1.**
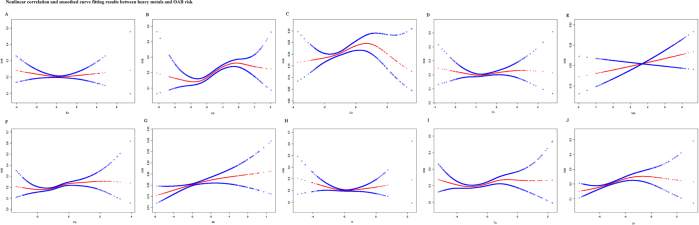
Nonlinear correlation and smoothed curve fitting results between heavy metals and OAB risk. Metal concentrations have been creatinine-corrected and ln-transformed, and analyses were adjusted for all covariates. (A) Ba; (B) Cd; (C) Co; (D) Cs; (E) Mo; (F) Pb; (G) Sb; (H) Tl; (I) Tu; and (J) Ur. OAB, overactive bladder.

Figure 1 demonstrates the nonlinear correlation and smoothed curve fitting results between ln-transformed, creatinine-corrected urinary metal concentrations and the possibility of OAB, after adjusting for all covariates. Figure 1A and H shows U-shaped associations between Ba and Tl concentrations and OAB risk, indicating negative correlations within specific ranges. Conversely, Pb, Sb, Tu, Ur, and Cd showed significant positive correlations with OAB risk. The curves for Ur and Cd versus OAB suggested potential saturation effects.

### Stratified analysis

**Figure 2.**
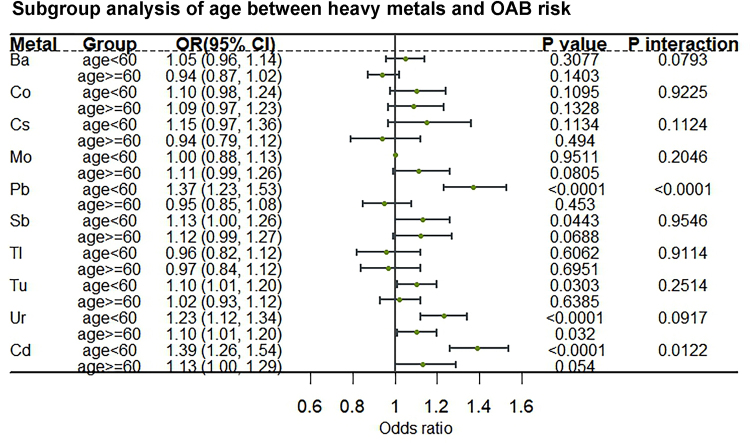
Subgroup analysis of age between heavy metals and OAB risk. OAB, overactive bladder.

**Figure 3.**
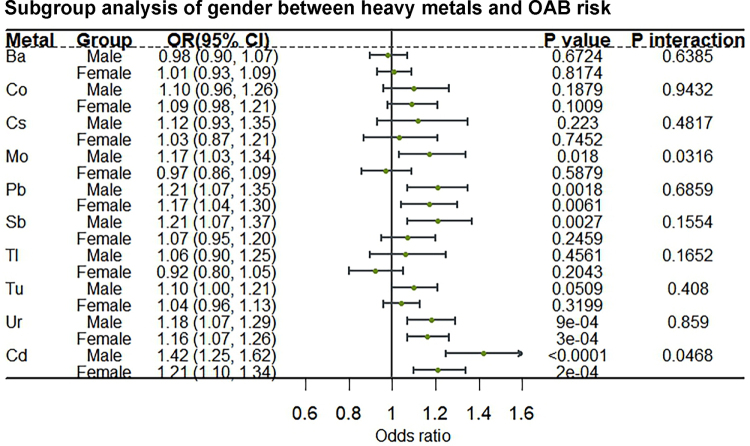
Subgroup analysis of gender between heavy metals and OAB risk. OAB, overactive bladder.

We performed stratified analyses and interaction tests by age and gender. Figure 2 shows the results of the stratified analysis with age ≥60 years subgroups. The association of Pb with increased OAB risk was significantly stronger in those aged <60 years than in those aged ≥60 years (OR 1.37, 95% CI 1.23–1.53 vs. OR 0.95, 95% CI 0.85–1.08), and the interaction was significant (*P* interaction <0.0001). The same trend was observed in the age stratification of Ur (OR 1.23, 95% CI 1.12–1.34 vs. OR 1.10, 95% CI 1.01–1.20). In the gender stratified analysis (Fig. 3), Cd had a significantly stronger positive association with OAB risk in males than in females (OR 1.42, 95% CI 1.25–1.62 vs. OR 1.21, 95% CI 1.10–1.34), and the interaction was significant (*P* interaction = 0.0468).

### WQS regression analysis

**Figure 4.**
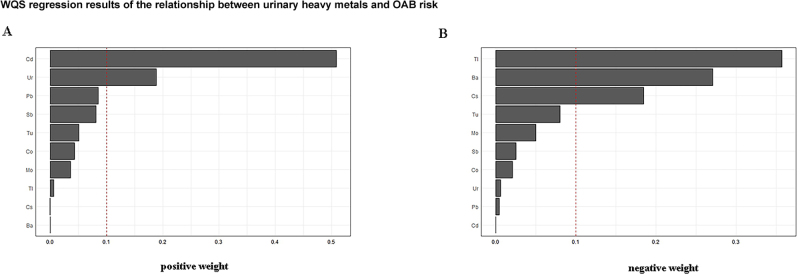
WQS regression results of the relationship between urinary heavy metals and OAB risk. (A) Positive direction, and Cd had the highest weight (0.497). (B) Negative direction, and the most dominant was Ba (0.447). OAB, overactive bladder; WQS, weighted quantile sum.

We explored the relationship between heavy metal mixtures and OAB risk by WQS regression analysis. When the mixture effect was constrained to be positive, OAB risk increased with increasing quartiles of the WQS index (OR 1.202, 95% CI 1.064–1.357). Figure 4A illustrates the estimated weights of each metal in the positive mixture index. Cd had the highest weight (0.497), followed by Ur (0.195) and Pb (0.090). When the mixture effect was constrained to be negative, an increase in the quartile of the WQS index was associated with a 14.3% reduction in OAB risk (OR 0.857, 95% CI 0.778–0.943). Ba had the highest weight (0.447), followed by Cs (0.211) and Tl (0.205) (Fig. 4B). The weights of each metal in the different models are detailed in Table 3.

**Table 3:** Summary of results from different models

Metal	Multivariate logistic regression	WQS positive direction	WQS negative direction	qgcomp
Cd	(+)	0.497	<0.001	0.5098
Ur	(+)	0.195	0.001	0.1337
Pb	(+)	0.090	0.005	0.1676
Sb	(+)	0.073	0.020	0.0214
Tu		0.051	0.049	0.0250
Co	(+)	0.047	0.011	0.1425
Mo		0.042	0.051	−0.0055
Tl	(−)	0.003	0.205	−0.2752
Cs		0.002	0.211	−0.1217
Ba	(−)	0.001	0.447	−0.5976

“(+)” means for positive correlation, “(−)” means negative correlation;

Ba, barium; Cd, cadmium; Co, cobalt; Cs, cesium; Mo, molybdenum; Pb, lead; Sb, antimony; Tl, thallium; Tu, tungsten; WQS, weighted quantile sum; Ur, uranium.

### qgcomp analysis

**Figure 5.**
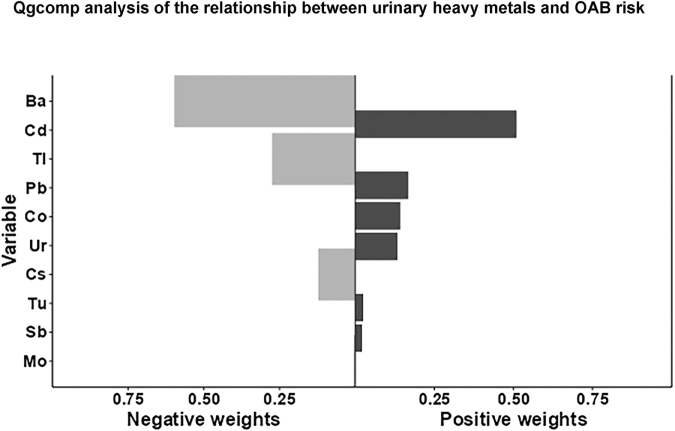
qgcomp analysis of the relationship between urinary heavy metals and OAB risk. OAB, overactive bladder.

The qgcomp analysis suggested an overall possible negative association between mixed heavy metal exposure and the risk of OAB (OR 0.4387, 95% CI 0.346–0.531). The estimated weights for each heavy metal are shown in Figure 5 and Table 3. Cd, Pb, Co, Ur, Tu, and Sb had positive weights, with Cd having the largest positive weight (0.5098). Ba, Tl, Cs, and Mo had negative weights, with Ba having the largest negative weight (0.5976).

### Sensitivity analyses

Multivariate regression models for males are presented in Supplemental Digital Content, Table 1, available at: http://links.lww.com/JS9/E902. A total of 1008 participants with complete information on OAB, heavy metals, and BPH were included in the analysis. The results showed that Ba was positively associated with OAB in model 1 without adjustment for covariates, whereas Pb, Sb, Ur, and Cd were negatively associated with OAB risk. Adjusting for BPH (model 2) did not substantially alter these findings. These correlations remained stable after treating heavy metals as categorical variables. Even after adjusting for all covariates, including BPH, Sb and Ur remained positively associated with OAB risk. Participants in the highest quartile of Sb and Ur had 1.69 (OR 1.69, 95% CI 1.01–2.84, *P* for trend = 0.0412) and 2.11 times (OR 2.11, 95% CI 1.25–3.56, *P* for trend = 0.0079) higher risk, respectively, compared to the lowest quartile. For females, a total of 3795 participants entered the analysis. The continuous variables Ba, Cs, and Tl were negatively associated, and Pb, Ur, and Cd were positively associated with OAB risk (Supplemental Digital Content, Table 2, available at: http://links.lww.com/JS9/E903). Adjusting for parity and vaginal delivery (model 2) did not markedly change these associations. After adjusting for all additional covariates, the risk of OAB increased by 13% for each unit increase in Ur (OR 1.13, 95% CI 1.02–1.24). These positive or negative correlations remained differentially significant after quartile categorization of heavy metals. Participants in the highest quartiles of Ur and Cd had a 40% (OR 1.40, 95% CI 1.08–1.81, *P* for trend = 0.0199) and 35% (OR 1.35, 95% CI 1.01–1.80, *P* for trend = 0.0219) higher risk of OAB, respectively, compared to the lowest quartile.

## Discussion

In this study, we investigated the association between 10 urinary heavy metals and the risk of OAB using NHANES data. To thoroughly evaluate the association between heavy metals and OAB, we assessed both the role of single metals and analyzed the overall role of metal mixtures while stratifying by the important variables. Our study found that there was a strong associations between urinary heavy metals and the risk of OAB among US adults; Cd, Pb, and Ur were the metals most strongly positively associated with OAB, with Cd having the strongest association; Ba, Cs, and Tl were the metals most strongly negatively associated, with Ba having the strongest negative association; the relationship between some heavy metals and OAB was nonlinear; and the strength of the correlation between heavy metals and OAB differed across age and gender subgroups.

OAB is a nonspecific disorder characterized primarily by urinary incontinence, a pathologic sensation that may be accompanied by overactivity of the detrusor muscles of the bladder^[31]^. The etiology of OAB is complex and varied, and central nervous system hypersensitivity may also play a role^[32]^. Recently, an association between MetS and OAB has been suggested. MetS may impair bladder function through an imbalance between the overproduction of oxidants and their elimination^[33]^. Oxidative stress, inflammation, and insulin resistance present in patients with MetS may cause or exacerbate OAB^[34]^. In addition, imbalances in circadian rhythm hormones and sex hormone secretion may also contribute to the development of OAB^[35]^. On the other hand, numerous studies confirm the positive correlation between heavy metals and MetS^[36–38]^ and suggest heavy metals may have an impact on the development of MetS through various mechanisms, such as their effects on oxidative stress and signaling^[38]^. A study using NHANES data from 2013 to 2016 reached that heavy metals were negatively correlated with male sex hormone-binding globulin (SHBG) and female estradiol levels^[39]^. Another review concluded that current evidence supports a correlation between metal exposure and hormone levels, but the direction of this correlation needs to be further investigated^[40]^. Cd and Pb exposures have been found to be positively associated with UUI in women^[12]^. A previous study investigated the relationship between blood Cd levels and the risk of OAB in a middle-aged and elderly population^[19]^. They reported a weak positive correlation between blood Cd levels and OAB, though statistically non-significant, association overall, but identified positive correlations with urinary incontinence severity in specific subgroups. This aligns with our findings. Our study analyzed the correlation between 10 creatinine-corrected urinary heavy metal concentrations and the risk of OAB. Our results revealed that Cd was consistently positively associated with OAB risk in multiple logistic regression, WQS regression, and qgcomp analysis, exhibiting the strongest positive weight. Another study explored the relationship between five blood heavy metals and OAB risk using data from NHANES 2011−2018^[41]^. They found a significant positive correlation between co-exposure to blood heavy metals and OAB risk, with Cd showing the strongest positive correlation. However, Ba and Tl were not included. Zhang *et al* found a positive correlation between urinary Cd and OAB, but analyzed only Cd and used fewer NHANES cycles^[42]^. To the best of our knowledge, no studies have directly explored the relationship between multiple urinary heavy metals and OAB. In our study, the correlation between urinary heavy metals and OAB was comprehensively evaluated using NHANES 2005–2018 data for both single metal and mixed exposures, and the complex effects of multiple metal exposures can be well explained.

Our study concludes that Cd and Pb are positively associated with OAB and ranked highest in importance for the positive mixture effect. It is well known that people are exposed to Pb and Cd through different pathways, including food, water, air, and tobacco^[43]^. In men, the correlation between Cd and Pb and SHBG remained significant after excluding the effects of other heavy metals^[44]^. In females, high Cd exposure may reduce hormone production and delay puberty, while high Pb exposure lowers inhibin B, a marker of follicular development^[45]^. In postmenopausal women, there is a significant negative correlation between Cd and serum estradiol and a positive correlation with testosterone^[46]^. Animal experiments suggest that estrogen may inhibit Rho-kinase function in bladder smooth muscle. In postmenopausal women, the incidence of urinary symptoms is higher due to estrogen withdrawal and decreased bladder detrusor contractility^[47]^. In addition, a study of 20 menopausal women showed that hormone therapy reduced autonomic and sensory nerve density^[48]^. These factors explain the increased incidence of OAB in sex hormone-deficient women. A growing body of research has demonstrated the efficacy of hormone therapy in postmenopausal women with OAB^[49]^. On the other hand, Cd may increase the risk of MetS by disrupting lipid metabolism^[50]^, generating reactive oxygen species, and inducing oxidative stress^[51]^. A Korean study found a higher risk of MetS in the highest Cd tertile concentrations^[52]^. Pb is also positively associated with MetS^[37]^. It has been suggested that patients with MetS have a higher incidence of OAB^[53]^. Animal studies indicate that MetS and estrogen hormone deficiency contribute significantly to the development of bladder voiding dysfunction via oxidative stress, apoptosis, mitochondrial dysfunction, increased bladder apoptosis, and interstitial fibrosis^[54]^, leading to OAB. Collectively, these findings support our conclusion that Cd and Pb may induce OAB through the disruption of metabolism and sex hormones. Additionally, Cd’s neurotoxicity^[55]^ could exacerbate OAB by damaging urinary regulatory nerves. Its nephrotoxicity, particularly in populations with metabolic diseases such as diabetes^[56]^, increases oxidative stress markers, potentially inducing or worsening OAB.

In our study, multivariate logistic regression analysis revealed that Ba was negatively correlated with OAB risk, and Ba dominated the negative mixture effect in WQS regression. As a common metal, Ba is widely used in the electronic industry, fireworks, and medical treatment^[57]^. However, nonlinear correlation analysis showed that the relationship between Ba and OAB was not purely linear, and there were inflection points of positive and negative correlation. Interestingly, the description of Ba in previous studies has also appeared to be two-fold. Some studies have suggested that Ba leads to a decrease in sex hormones and an increased incidence of MetS^[57,58]^, while others show positive correlations between Ba and sex hormones^[59]^. This suggests that the relationship between Ba and hormones, and even OAB, may not be linear. Considering Ba2+ promotes vascular smooth muscle contraction^[60]^, we speculate it may similarly affect bladder smooth muscle. High urinary Ba levels may reflect lower systemic Ba^2^⁺, potentially diminishing its contractile effect on the detrusor muscle and reducing involuntary contractions characteristic of OAB^[1]^. This may explain the observed negative association.

Our study has notable strengths. This study explored the correlation between heavy metals and OAB risk using a variety of statistical methods, not only analyzing the effects of single metals but also evaluating the effects of mixtures. As far as we know, no other research has identified the association between heavy metals and OAB thus far. This provides a theoretical basis for environmental and health-related policymakers. They are reminded that they should pay sufficient attention to heavy metal concentrations in the environment and set monitoring thresholds to minimize the multiple health effects^[61]^. It also offers insights for OAB management and prevention. Specifically, testing urinary heavy metal levels provides clinicians with a noninvasive and convenient approach to assess potential OAB risk factors. Urinary heavy metal detection could aid clinicians in OAB diagnosis and subsequent management strategies, potentially reducing the societal burden of OAB. However, limitations of this study are inevitable. First, the nature of cross-sectional studies makes it difficult to draw precise inferences about the causal association between heavy metal exposure and OAB risk. Second, there is no standardized time of urine collection in the NHANES database, and heavy metal concentrations in urine may vary over time^[62]^. Although heavy metal concentrations are creatinine-corrected, this may still result in erroneous measurements of urinary heavy metals that could affect the assessment of OAB risk. Finally, the study population was American, and the findings may not be applicable to other countries and regions. In the future, more longitudinal studies exploring the correlation between heavy metals and OAB risk are clearly necessary. Nevertheless, our findings significantly contribute reliable evidence on the heavy metal–OAB risk association.

## Conclusion

In summary, our study confirms that urinary heavy metals are significantly associated with OAB risk among US adults. Cd exhibits the strongest positive association, while the largest negative correlation with OAB risk is Ba. The relationship for some heavy metals is not linear. Future prospective studies with more comprehensive and larger sample sizes are needed to assess the effects of single or mixed exposure to heavy metals on OAB risk.

## Data Availability

The data were obtained from the official NHANES website (https://www.cdc.gov/nchs/nhanes/) and are completely public and free.
